# Putting the human into health systems: achieving functional integration of service delivery in Kenya and Swaziland

**DOI:** 10.1186/1472-6963-14-S2-P75

**Published:** 2014-07-07

**Authors:** Susannah Mayhew, Richard Mutemwa, Manuela Colombini, Martine Collumbien

**Affiliations:** 1 Department of Global Health and Development, London School of Hygiene & Tropical Medicine, London, UK; 2 Department of Social & Environmental Health Research, London School of Hygiene & Tropical Medicine, London, UK

## Background

The Integra Initiative has evaluated different models of integrating FP/PNC and HIV testing and treatment services in Kenya and Swaziland. Human and physical resource integration (“structural” integration) is the usual outcome measure of “successful” service integration. Integra research has shown that in fact functional integration (clients actually receiving integrated care) does not necessarily follow. Much depends on the actions of individual providers and their managers in combination with systems and other factors. This paper provides a meta-analysis from a range of Integra data to investigate the human factors influencing successful “functional” service integration.

## Materials and methods

Integra is a multi-method, non-randomized, pre-post intervention trial. This presentation draws on three sets of data from 42 facilities in Kenya and Swaziland:

1) structured surveys with providers analysed using Stata 11.2;

2) in-depth interviews with 56 providers coded using Nvivo 8; analyzed using thematic analysis.

3) “contextual” data that were collected on other activities at study facilities relating to integration that occurred during the study period.

By triangulation of the data, we present case studies of successful clinics to analyze factors facilitating successful functional integration.

## Results

Qualitative and quantitative data indicate that providers at high-functioning integrated clinics perceived improved technical quality, service efficiency and cost-benefits but also noted increases in workloads, less quality time with clients and occupational stress. Despite the challenges, many providers found ways to cope - through better team-working and load-sharing which facilitated better integration. Providers valued skills enhancement, more variety and challenge in their work and better job satisfaction through increased client-satisfaction.

Staff numbers are a critical issue but also how roles are shared between them as well as the relevance of their skills to their allotted tasks. Qualitative data further highlight the importance of facility management, supervisory support and innovation for on the job training and mentorship of providers. Contextual data show how donor/NGO activities and unilateral actions by facility managers can both support and impede integration.

## Conclusion

Most staff are supportive of integration but formal support mechanisms are needed to help providers cope with high stress and manage increased workloads. Lessons can be learned from providers reporting good teamwork to cope with increased workload and waiting times. Achieving functional integration requires attention to the structures and mechanisms in place to support both managers and frontline providers. Consultation with health care workers themselves is essential to improve integrated health systems that are people centred.

